# Mr. Wawn, on the Croup

**Published:** 1804-03-01

**Authors:** Charles N. Wawn

**Affiliations:** No. 48, Poland Street


### *To the Editors of the Medical and Physical Journal.*

Gentlemen,

I AM induced to trouble you with the following remarks on a peculiar plan of treatment of Croup, from a hope, that through the publicity and extensive circulation of your valuable Journal, a remedy which promises considerable success, and which does not appear to have been hitherto known or appreciated in proportion to its merits, may be brought before the medical public, with a view to ascertain and establish its apparently intrinsic worth. The exhibition of calomel, as a curative means in Croup, is not new; but its use appears to have been confined to individuals, or particular places. Mr. James Anderson, sen. of Edinburgh, was, I believe, the first who publicly noticed it, in a paper on the subject in Dr. Duncan's Annals of

Medicine for 1799. He exhibited it on the authority of a physician of eminence, who had heard of its efficacy in this disease in America; and in four cases he states, it appears to have been decidedly beneficial. The utility of it he further confirms in a subsequent paper in the above-mentioned work for 1801, where he also mentions his having found it requisite to administer it more liberally in urgent cases.

Mr. Rumsey, a surgeon in Buckinghamshire, has inserted in a work, entitled, "Transactions of the London Society for improving Medicine and Surgery," an ingenious paper on Croup, in which several cases are related, successfully treated by calomel; he ingenuously owns, however, that in two instances which occurred to his brother, it was given without good effect; but its failure of success in these, if treated with a proportionate quantity of mercury to that given by Mr. Rumsey, I should be much disposed to attribute to the insufficiency of the dose, and the too long intervals suffered to elapse between each exhibition. In two confirmed cases, to be noticed hereafter, much larger quantities of the medicine were given; and after considering the rapid progress of inflammation in the trachea here, the increase of danger from every delay in the use of the most active remedies, and the large quantity of mercury that children will bear without producing any sensible effect, I cannot help thinking its use might be safely extended. Dr. Cheyne, in Essay 1, (on Croup) of his splendid work on the diseases of children, observes, that "it has been proposed to give children calomel under this disease, throwing it in quickly with a view to produce salivation—that he had ordered it in the second stage but never found it of any service." In most of those cases I have heard of being cured by it, no degree of ptyalism was brought on, nor any obvious effect, except that of powerfully debilitating, and producing the singular phenomenon of dark green stools, the appearance of which has by some been regarded as the criterion for desisting from the further use of the remedy. Several interesting cases of cynanche trachealis, successfully treated by this plan, were related at the meetings of the Lyceum Medicum Londinense during the last session, by Mr. Robinson, an ingenious practitioner then in the army; and his evidence in favour of this medicine was corroborated by an obliging letter from Dr. Hamilton of Edinburgh, who had also exhibited it with great success. Mr. Thomas, of Leicester Square, a gentleman of known science and abilities, also mentioned mentioned a case, at one of the present meetings of the Lyceum, which had just occurred to him, and in which he had effected a cure by the free use of the same medicine: To a child of 12 months old, six grains of calomel were at first given, and repeated in four hours, and two grains every two hours after, till thirty grains had been taken; when the peculiar appearance in the stools was produced, the medicine was desisted from, and the child recovered.

I shall now beg leave to conclude this paper, already I fear too long, with the narration of a well marked case which has recently fallen under my own observation, and which I attended for Mr. Newby of Poland Street, who was at that time confined in consequence of a domestic calamity. A robust child of seventeen months old, was attacked with cough, cold, and fever, which were quickly succeeded with symptoms of croup. The friends of the child, not aware of the insidious nature of the disease, deferred applying for advice till after a lapse of two days, when I found it labouring under pyrexial symptoms, quickened and laborious breathing with a highly flushed countenance; but what immediately struck me was the peculiar shrill noise accompanying each inspiration, and the rough stridulous cough which so unequivocally characterize the cynanche trachealis. The pulse was frequent and full; bowels regular. Five grains of calomel were ordered to be given immediately in a little thick syrup, and two grains every two hours after till some obvious alteration should take place, and in addition to these four leeches to be applied to the sternum. In the evening I found my little patient evidently relieved; eleven grains of calomel had been given with very little effect on the bowels; respiration was more moderate and natural; cough less urgent; the force and frequency of arterial action much diminished, and the face not so florid. The calomel was ordered to be continued in doses of two grains every two hours as before, unless excessive purging should be induced, and a blister to be applied to the breast. Next morning I found so great an abatement of all the symptoms, as to be able to pronounce the child out of immediate danger; twelve grains more of calomel had been given, and had produced several loose stools, some of which were of a dark green colour, and contained a number of films like threads of coagulated lymph; the inordinate action of the heart and lungs had now subsided, and nothing but a croupy cough remained. On inquiring if any portions of a membrane had been coughed up, the mother of the child informed me she had twice observed its mouth covered with a large white skin after a fit of coughing, but before she could succeed in her attempts to extricate it with a spoon handle the child had swallowed it. With a view to detach any portions of the membrane that might be left, I advised a smart emetic, but notwithstanding nearly one scruple of ipecac. and four grains of tartarized antimony were given at short intervals, little vomiting was produced, and that without any appearance of skins. From this time the remains of the complaint made a gradual retrocession, and the child recovered.

*No.* 48, *Poland Street,* I am, &c.

*Feb.* 10, 1804. CHARLES N. WAWN.

